# Supplementary figures and images for: Characterization of full-length CNBP expanded alleles in myotonic dystrophy type 2 patients by Cas9-mediated enrichment and nanopore sequencing
Source: eLife. 2022 Aug 26;11:e80229. doi: 10.7554/eLife.80229 (PMC9462847; doi:10.7554/eLife.80229)

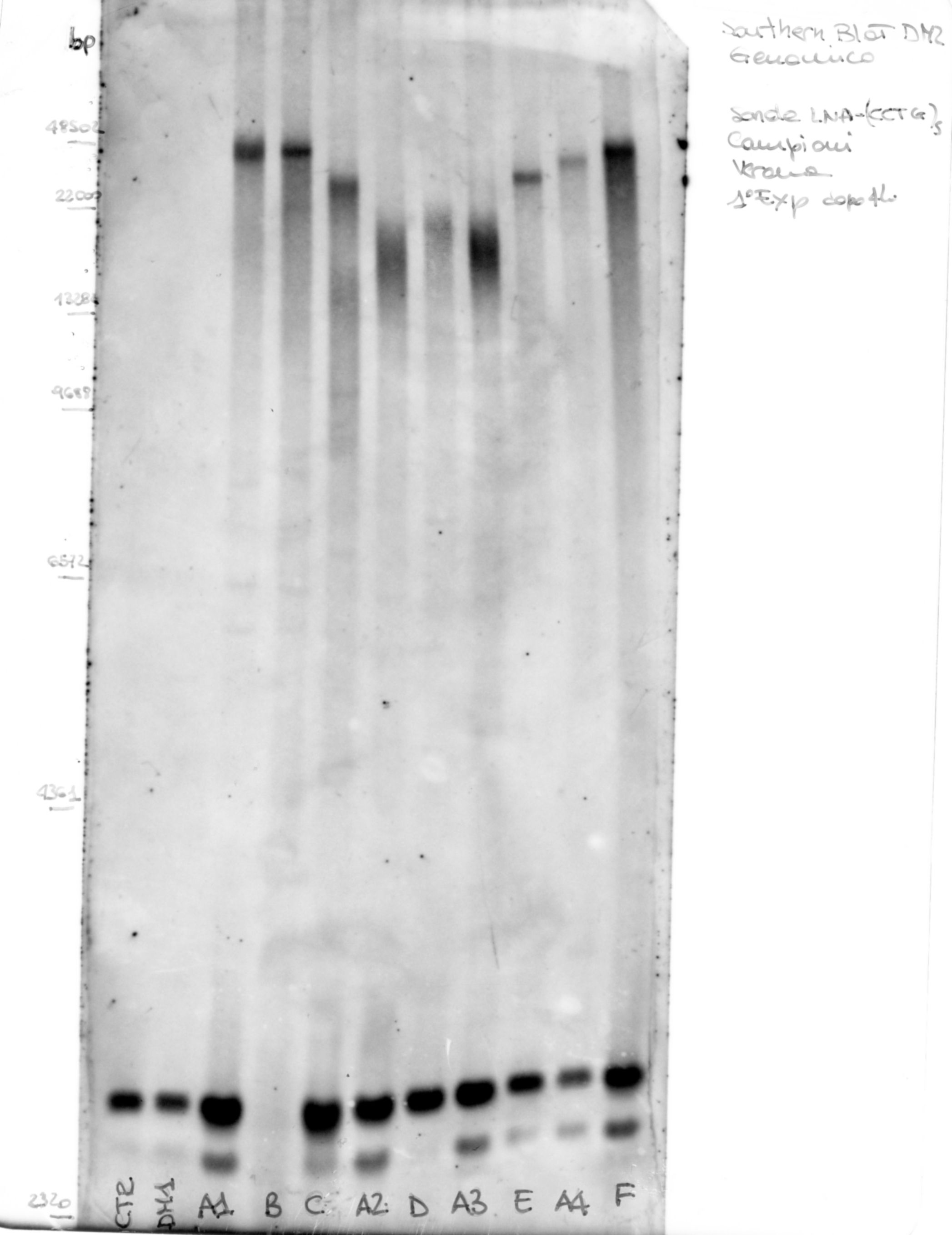

Supplement: Figure 1—figure supplement 2—source data 1. [file elife-80229-fig1-figsupp2-data1.zip › Figure 1_figure supplement 2_source data 1.jpg]

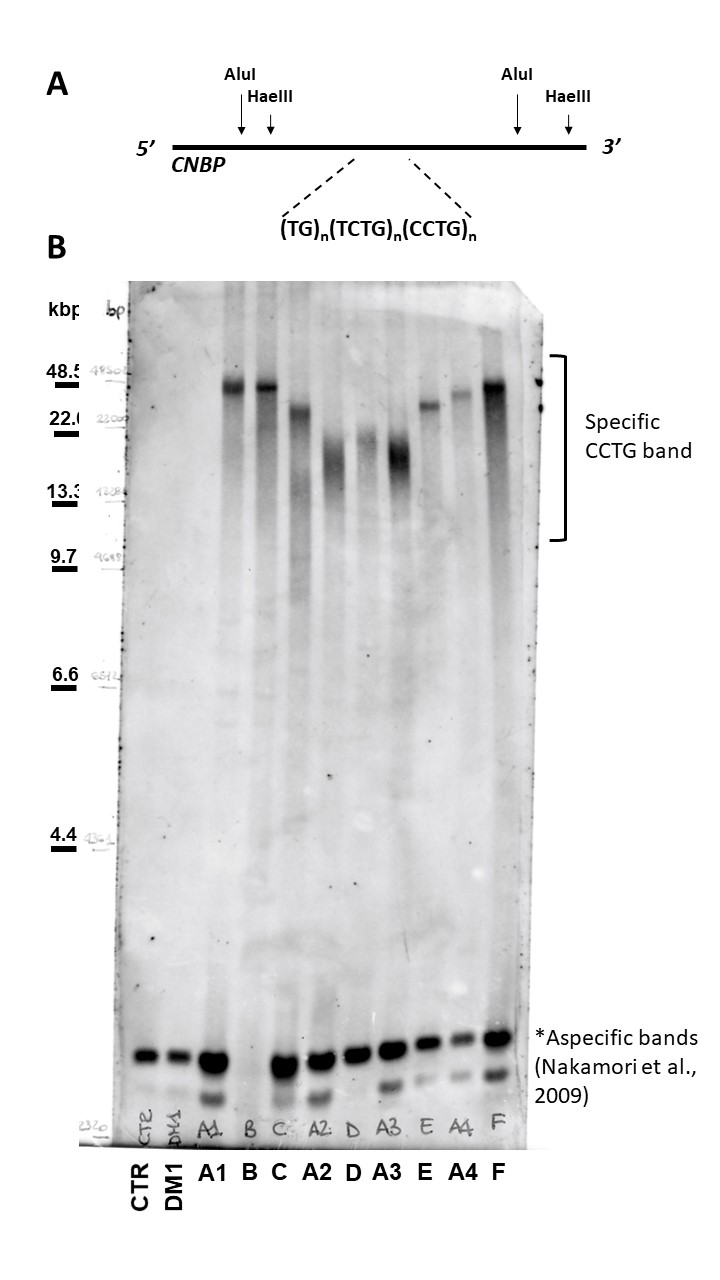

Supplement: Figure 1—figure supplement 2—source data 2. — (A) Restriction map of the dystrophy type 2 (DM2) locus indicating the AluI and HaeIII restriction sites used for the digestion of genomic DNA. (B) Southern blot analysis of genomic DNA double digested with AluI and HaeIII and probed with a digoxigenin (DIG)-labeled (CCTG)5 locked nucleic acid (LNA) probe. Lane 1, CTR, healthy control sample; lane 2, DM1 sample; lanes 3–11, DM2 samples. Molecular markers are indicated on the left. *indicates aspecific bands, as reported by Nakamori et al., 2009. [file elife-80229-fig1-figsupp2-data2.zip › Figure 1_figure supplement 2_source data 2.jpg]
