# Supplementary material for: Characterization of full-length CNBP expanded alleles in myotonic dystrophy type 2 patients by Cas9-mediated enrichment and nanopore sequencing
Source: eLife. 2022 Aug 26;11:e80229. doi: 10.7554/eLife.80229 (PMC9462847; doi:10.7554/eLife.80229)
Supplement: Supplementary file 1. — The table reports the feature of each Cas9-mediated sequencing experiment performed and the sequencing statistics of each ONT run. Average values are also provided for singleplex and multiplex runs separately. [file elife-80229-supp1.docx]

**Supplementary File 1. Sequencing statistics of singleplex and multiplex experiments.** The table reports the feature of each Cas9-mediated sequencing experiment performed and the sequencing statistics of each ONT run. Average values are also provided for singleplex and multiplex runs separately.

| **Run ID** | **Exp1_Singleplex** | **Exp2_Singleplex** | **Exp3_Singleplex** | **Exp4_Singleplex** | **Exp1_Multiplex** | **Exp2_Multiplex** | **Exp3_Multiplex** | **Exp4_Multiplex** |
| --- | --- | --- | --- | --- | --- | --- | --- | --- |
| **Source** | Whole blood | Whole blood | Whole blood | Whole blood | Whole blood | Whole blood | Whole blood | Whole blood |
| **# samples** | 1 | 1 | 1 | 1 | 5 | 4 | 4 | 3 |
| **Sample ID** | A2 | E | F | B | A1, A3, A4, C | A1, A3, A4, C | A4, B, D | B, D, F |
| **Input DNA** | 5 ug | 2 ug | 6,8 | 4,6 | 2 ug / sample | 1 - 4 ug / sample | 3 - 5 ug / sample | 4.5 - 10 ug / sample |
| **Total reads** | 186.622 | 40.122 | 27.693 | 92.124 | 1.504.794 | 917.260 | 114.967 | 653.967 |
| **Total aligned PASS reads** | 154.046 | 35.025 | 21.550 | 60.315 | 1.284.125 | 819.341 | 98.083 | 552.544 |
| **On-target PASS reads On CNBP** | 624 | 283 | 330 | 142 | 417 | 397 | 75 | 501 |
| **On-target reads %** | 0,41% | 0,81% | 1,53% | 0,24% | 0,03% | 0,05% | 0,08% | 0,09% |
| **On-target avg. cov. (X)** | 584,2 | 279,8 | 311,9 | 127,6 | 507,81 | 357,54 | 78,35 | 494,44 |
| **Whole genome avg. cov. (X)** | 0,14 | 0,077 | 0,07 | 0,07 | 0,94 | 0,6 | 0,118 | 0,66 |
| **Fold enrichment** | 4.173 | 3.634 | 4.456 | 1.823 | 540 | 596 | 664 | 749 |
|  |  |  |  |  |  |  |  |  |
|  |  |  |  |  |  |  |  |  |
|  | **Average values** | |  |  |  |  |  |  |
|  | **Singleplex (N=4)** | **Multiplex (N=4)** |  |  |  |  |  |  |
| **Total aligned reads** | 67734 | 688523 |  |  |  |  |  |  |
| **On-target reads** | 345 | 348 |  |  |  |  |  |  |
| **On-target %** | 0,01 | 0,001 |  |  |  |  |  |  |
| **On-target avg. cov. (X)** | 325,9 | 359,5 |  |  |  |  |  |  |
| **Whole genome avg. cov. (X)** | 0,1 | 0,6 |  |  |  |  |  |  |
| **Fold enrichment** | 3521,3 | 637,3 |  |  |  |  |  |  |
